# Direct Synthesis of Mn_3_[Fe(CN)_6_]_2_·nH_2_O Nanosheets as Novel 2D Analog of Prussian Blue and Material for High-Performance Metal-Ion Batteries

**DOI:** 10.3390/mi14051083

**Published:** 2023-05-21

**Authors:** Artem A. Lobinsky, Maria V. Kaneva, Maxim I. Tenevich, Vadim I. Popkov

**Affiliations:** Ioffe Institute, 194021 Saint-Petersburg, Russia

**Keywords:** MnFCN, Prussian blue analogue, 2D materials, nanosheets, SILD, metal-ion batteries

## Abstract

Rechargeable metal-ion batteries (RMIBs) are prospective highly effective and low-cost devices for energy storage. Prussian blue analogues (PBAs) have become a subject of significant interest for commercial applications owing to their exceptional specific capacity and broad operational potential window as cathode materials for rechargeable metal-ion batteries. However, the limiting factors for its widespread use are its poor electrical conductivity and stability. The present study describes the direct and simple synthesis of 2D nanosheets of MnFCN (Mn_3_[Fe(CN)_6_]_2_·nH_2_O) on nickel foam (NF) via a successive ionic layer deposition (SILD) method, which provided more ion diffusion and electrochemical conductivity. MnFCN/NF exhibited exceptional cathode performance for RMIBs, delivering a high specific capacity of 1032 F/g at 1 A/g in an aqueous 1M NaOH electrolyte. Additionally, the specific capacitance reached the remarkable levels of 327.5 F/g at 1 A/g and 230 F/g at 0.1 A/g in 1M Na_2_SO_4_ and 1M ZnSO_4_ aqueous solutions, respectively.

## 1. Introduction

Over the last few years, significant attention has been directed towards developing diverse categories of metal-ion batteries. Sodium- and potassium-ion batteries have gained significant attention in light of their extensive utilization and cost-effective nature as well as their superior safety during operation when contrasted with lithium-ion batteries. In addition, recently, metal-ion batteries that use two-charge cations (Zn^2+^, Mg^2+^, Ca^2+^, etc.) have attracted much attention since they have a higher volume energy density due to the amount of charge that is transferred [1,2,3]. Of particular interest are zinc-ion batteries with aqueous electrolytes since they can be used to create compact, safe, and inexpensive autonomous power sources with large specific capacities. 

The rechargeable zinc-ion batteries demonstrate the potential to satisfy the criteria of contemporary electric energy storage systems owing to abundant zinc-based resources, economic feasibility, and their substantial theoretical specific capacity (up to 820 mAh/g [4]). Therefore, these batteries have emerged as a promising substitute for lithium-ion batteries. Recently, certain achievements have been observed that improved the electrochemical parameters of electrode materials for such devices, but researches have not identified suitable cathode materials that can withstand multiple cycles of intercalation/deintercalation of zinc ions [5]. The stability of a zinc-ion battery’s structure depends greatly on the cathode material’s ability to chemically bond with Zn^2+^ ions quickly and efficiently while maintaining a strong and durable structure. Therefore, features such as a crystal framework that has appropriate crystallographic sites for the placement of Zn^2+^, pathways for ion diffusion that require minimal energy, and efficient electronic conductivity for the transport of charges are favorable for the cathode. Often, compounds with a layered tunnel structure and redox components meet these requirements. Consideration should also be given to the porosity of the cathode, which enables the infiltration of the electrolytic solution, thereby intensifying the number of active sites and the maximum rate of charge transfer [6]. From this point of view, Prussian blue analogues and oxides of manganese, vanadium, and cobalt as well as some organic compounds can be used as cathode materials. Among these materials, Prussian blue analogues (hexacyanoferrates) are the most promising for further commercialization due to their wide operating potential window and high specific capacity. Additionally, their synthesis can be achieved by employing comparatively cost-effective and readily available precursors [7,8,9]. It was previously discovered that manganese (II) hexacyanoferrates (III) Mn_3_[Fe(CN)_6_]_2_ with the elimination of interstitial water gave an increased reversible capacity and enhanced cycle stability [10]. However, the practical values of the specific capacitance are often far from the theoretical values due to the low electronic conductivity of these cathode materials. The use of two-dimensional nanocrystals with a “nanosheets”-like morphology, which have a set of distinctive physical and chemical characteristics, will solve this problem. The planar two-dimensional morphology of these materials affords a suitable quantity of effective adsorption sites. One salient attribute of two-dimensional (2D) materials pertains to their exceedingly slender dimensions, typically spanning a few nanometers. This property enables charge carriers to traverse minuscule distances from the material volume to its surface, leading to a substantial enhancement of electronic conductivity [11]. As noted in the above-mentioned works, a significant challenge in the development of cathode materials for hybrid batteries lies in the intricacy of attaining ultrathin nanocrystals exhibiting the graphene-like morphology of “nanosheets” through existing synthesis techniques. Such a material morphology holds potential for mitigating electroactive material degradation and enhancing electronic conductivity.

To solve the above-mentioned problems in the synthesis of electrode materials with 2D morphologies, the most promising approach, from our point of view, is the use of layer-by-layer synthesis methods, also known as successful ionic layer adsorption and reaction (SILAR) or successive ionic layer deposition (SILD) [12]. The SILD method allows the regulation of a wide range of synthesis conditions (the number of processing cycles, the processing sequence, the processing time in reagent solutions, the concentration, the anionic composition and pH of reagent solutions, the heating of the obtained samples, etc.) to influence the morphology, composition, and structure of the synthesized compounds as well as to obtain various 1D, 2D, and 3D nanomaterials, which is important for the creation of highly effective electroactive materials [13,14,15,16]. A considerable body of research exists on the subject of the layer-by-layer synthesis of transition metal oxides for application in electrode materials, including those intended for utilization in zinc-ion batteries [17,18,19,20,21].

In this work, we describe a simple and direct technique for producing 2D Mn_3_[Fe(CN)_6_]_2_·nH_2_O nanosheets using successive ionic layer deposition (SILD) from an aqueous solution of MnSO_4_ and K_3_[Fe(CN)_6_] and an investigation of its electrochemical performances in different types of electrolytes. The obtained Mn_3_[Fe(CN)_6_]_2_·nH_2_O nanosheets are examined as cathode materials for metal-ion batteries and supercapacitors as a proof-of-concept application.

## 2. Materials and Methods

### 2.1. Materials

Highly pure analytical-grade reagents were used for synthesis: manganese sulfate pentahydrate (MnSO_4_·5H_2_O), potassium ferrocyanide (K_3_[Fe(CN)_6_]), hydrochloric acid (HCl), and potassium hydroxide (KOH). All reagents were produced by Vecton (Russia). Nickel foam (110 PPI; size: 20 × 30 mm) was used as a carrier.

### 2.2. Synthesis of Mn_3_[Fe(CN)_6_]_2_ Nanosheets

The nickel foam underwent pre-treatment with acetone, 3 M hydrochloric acid, and deionized water. For the preparation of a coating of Mn_3_[Fe(CN)_6_]_2_·nH_2_O (denoted further as MnFCN) on the Ni foam using the SILD method, two aqueous precursor solutions were used. The optimal synthesis conditions (the concentration of the precursor solutions (0.01 M), the processing time in the precursor solutions (30 s), and the number of treatment cycles (50 cycles)) were determined in accordance with the methodology [12]. Under these conditions, the formation of a uniform coating of two-dimensional structures of Mn_3_[Fe(CN)_6_]_2_·nH_2_O on the surface of the nickel foam occurred. 

The cationic precursor was a 0.01 M MnSO_4_ solution, and a 0.01 M K_3_[Fe(CN)_6_] solution was used as the anionic precursor. The SILD cycle consisted of four stages: the nickel foam was immersed in the cationic precursor for 30 s (1); the excess reagent was removed by rinsing the nickel foam with deionized water for 30 s (2); after that, the nickel foam was immersed in the anionic precursor for 60 s (3); and the excess reagent was removed again by rinsing the nickel foam with deionized water for 30 s (4). This SILD cycle was repeated 50 times. Reactions between adsorbed Mn^2+^ cations and adsorbed [Fe(CN)_6_]^3−^ anions led to the formation of a MnFCN thin film. After SILD synthesis, the nickel foam was air-dried at an ambient temperature.

### 2.3. Material Characterization

The MnFCN thin film was characterized using scanning electron microscopy (SEM) on a Tescan Vega 3 SBH microscope (Tescan, Brno, Czech Republic) to reveal its surface morphology, using an energy-dispersive X-ray (EDX) spectrometer attached to an Oxford INCA 350 (Oxford Instruments, Abingdon, UK) to detect the elements and the surface distribution of Mn and Fe, and using X-ray diffraction (XRD) with Cu Kα radiation (λ = 0.1542 nm) on a Rigaku SmartLab 3 Powder X-ray diffractometer (Rigaku, Tokyo, Japan) to display the crystalline phases. The qualitative and quantitative analysis of the samples was characterized via X-ray photoelectron spectroscopy (XPS) using an ESCALAB 250Xi electron spectrometer (ThermoFisher Scientific, Waltham, MA, USA).

### 2.4. Electrochemical Characterization

The electrochemical activity of the prepared MnFCN was tested in a three-electrode system containing MnFCN, which was deposited on the Ni foam (with a mass loading of 1.2 mg/cm^2^) and served as the working electrode; Ag/AgCl (3 M KCl) or Hg/HgO (1 M NaOH), which were used as reference electrodes; and a graphite rod, which was used as the counter electrode. Cyclic voltammetry (CV), electrochemical impedance spectroscopy (EIS), and galvanostatic charging/discharging (GCD) measurements were performed using an Elins P-45X electrochemical workstation with an FRA24M module of electrochemical impedance (Chernogolovka, Russia) in different electrolytes (1 M NaOH, 1 M Na_2_SO_4_, and 1 M ZnSO_4_ aqueous solutions). CV measurements were obtained in the potential windows from 0.0 V to 1.2 V (vs. Ag/AgCl) or from 0.0 V to 0.5 V (vs. Hg/HgO) at different scan rates (2–20 mV/s), and GCD measurements were obtained at various current densities (0.1–5 A/g). EIS measurements were obtained at the open-circuit potential (0.224 V vs. Ag/AgCl) within a frequency range from 50 kHz to 0.1 Hz and a sinusoidal wave with a 5 mV amplitude. The specific capacitance (C, F/g) of the prepared MnFCN was calculated from the GCD measurements using Equation (1):

(1) $$C=\frac{Idt}{dVm}$$

where dV is the potential window (V), m is the mass of the MnFCN deposited on the Ni foam (g), I is the applied current (A), and dt is the discharge time (s) [22]. The mass of the MnFCN (2.8 mg) electroactive materials was measured using a JOANLAB FA1204 microbalance (China).

## 3. Results and Discussion

**Figure 1:**
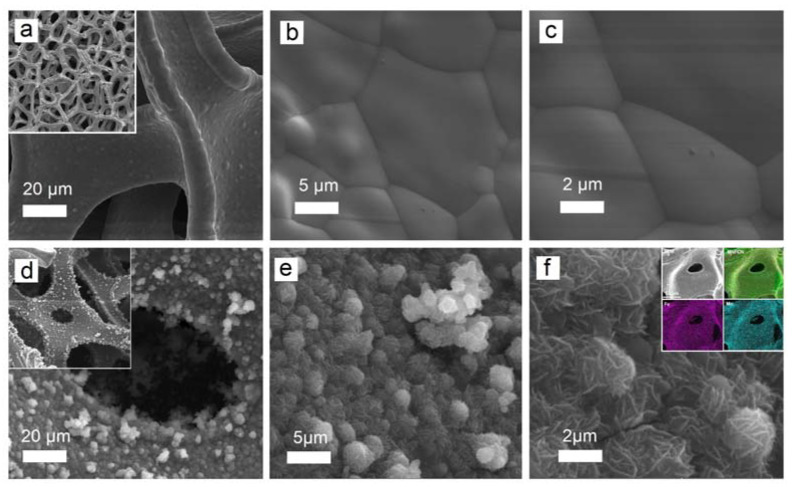
SEM images of nickel foam carrier (**a**–**c**) and MnFCN coating (**d**–**f**) with different magnifications (EDX elemental mapping of MnFCN coating is shown in inset of (**f**)).

Figure 1a–c show the surface of a nickel foam carrier at different magnifications. Figure 1d–f show MnFCN nanostructures grown on nickel foam. As can be seen, individual globules (2–6 µm) are formed by a set of 2D nanosheets (10–20 nm) (Figure 1d). The morphology of MnFCN in the form of 2D nanosheets has the potential to enhance the properties of the electrode surface, facilitate charge transport, and improve the consistency of the electrochemical performance of the electrode specimen. The MnFCN’s nanostructure, consisting of multiple layers, facilitates more efficient electrochemical reactions and promotes a greater active electrode surface area, thereby enhancing the overall performance of the material. The energy-dispersive X-ray spectroscopy (EDX) elemental mapping results are shown in the inset of Figure 1f. The elemental analysis results indicate the existence of the elements Mn, Fe, C, and N. A homogenous distribution of Mn and Fe atoms is observable over the entirety of the surface of the nickel foam (Figure 1f inset).

**Figure 2:**
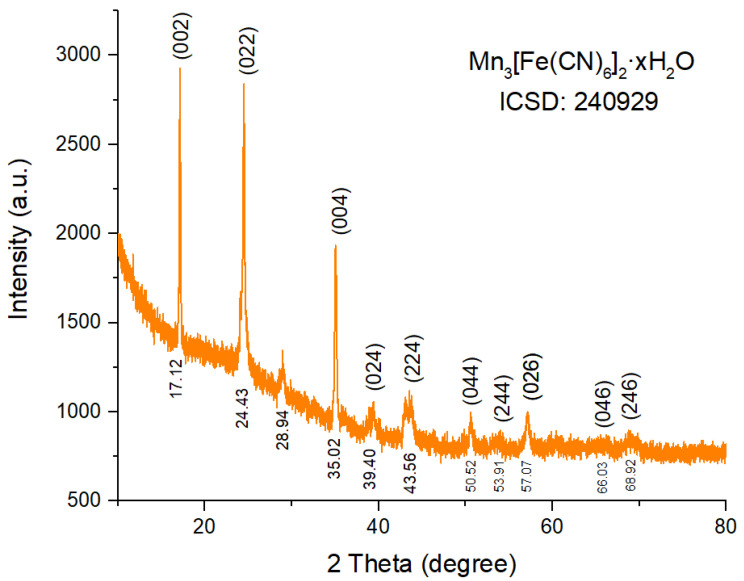
XRD pattern of MnFCN.

The crystalline phase structure of MnFCN was analyzed using X-ray diffraction (XRD), as depicted in Figure 2. The XRD pattern of MnFCN contains characteristic peaks at 17.12°, 24.43°, 35.02°, 39.40°, 43.56°, 50.52°, 53.91°, 57.07°, 66.03°, and 68.92° associated with planes (002), (022), (004), (024), (224), (044), (244), (026), (046), and (246), respectively. The observed characteristic peaks are in full agreement with the structure of Mn_3_[Fe(CN)_6_]_2_·nH_2_O (ICSD card #24-00929 [23]).

**Figure 3:**
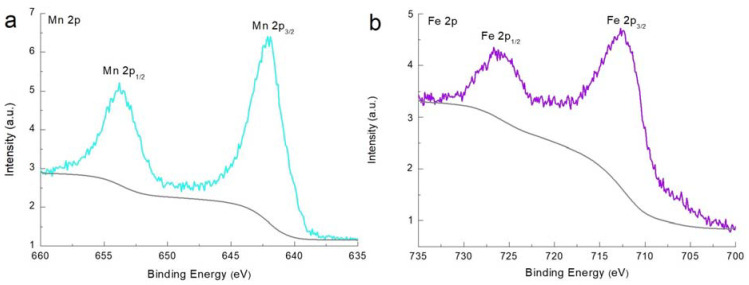
XPS spectra of (**a**) Mn 2p and (**b**) Fe 2p of MnFCN.

X-ray photoelectron spectroscopy (XPS) was conducted to analyze the chemical compounds and their corresponding oxidation states. The Mn 2p XPS spectrum of MnFCN is shown in Figure 3a. The Mn 2p spectrum showed two peaks at 642.0 and 653.8 eV, which corresponded to the Mn 2p_2/3_ and Mn 2p_1/2_ orbitals and could be fitted to the peaks ascribed to the Mn^2+^ state [24]. Figure 3b shows the XPS spectrum of Fe 2p. The Fe 2p peaks of MnFCN were observed at 712.4 and 726.2 eV and were attributed to Fe 2p_3/2_ and Fe 2p_1/2_. This revealed that Fe exists in the Fe^3^+ state in MnFCN [24]. These results were in good agreement with the EDX data. Therefore, the molecular formula of the final compound was established as Mn_3_[Fe(CN)_6_]_2_·nH_2_O.

The electrochemical characteristics of an NF electrode that comprised nanolayers of Mn_3_[Fe(CN)_6_]_2_·nH_2_O (MnFCN/NF) were investigated via various methods such as CVA, GCD, and EIS using different types of electrolytes.

**Figure 4:**
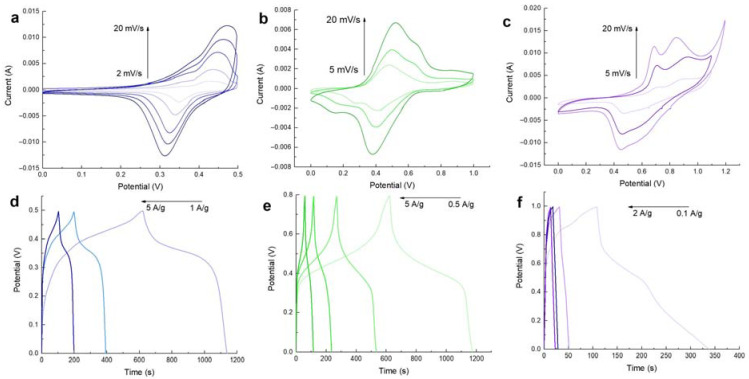
CVA and CD curves of MnFCN/NF in (**a**;**d**) 1M NaOH, (**b**;**e**) 1M Na_2_SO_4_, and (**c**;**f**) 1M ZnSO_4_ electrolytes, respectively.

Electrochemical measurements of MnFCN/NF as a cathode for a pseudocapacitor were obtained using 1 M NaOH. Figure 4a shows CVA curves of MnFCN/NF in the range from 0.0 V to 0.5 V vs. Hg/HgO. On the CVA curves, one pair of wide redox peaks, which possibly originated from the fast redox reactions of Mn^2+^ → Mn^4+^ and Mn^4+^ → Mn^2+^ on the electrode/electrolyte interface, suggests the capacitive behavior of this material. Figure 4d depicts the galvanostatic charge/discharge profiles of the MnFCN/NF electrode at varying current densities (1, 2, and 5 A/g). The MnFCN/NF electrode’s specific capacitance was determined using Equation (1) and was found to be 1032 F/g at 1 A/g in an alkaline medium (1M NaOH).

The cyclic voltammetry curves (CVA) of MnFCN/NF electrodes, which were obtained at varying scan rates within a 1 M Na_2_SO_4_ electrolyte, are displayed in Figure 4b. The obtained results indicate the presence of two sets of clearly observable redox peaks. The initial set of redox peaks noted at 0.55 V and 0.4 V vs. Ag/AgCl are indicative of redox manganese reactions involving Mn^2+^ → Mn^3+^ and Mn^3+^ → Mn^2+^, respectively. The second set of redox peaks, occurring at potential values of 0.72 V and 0.23 V vs. Ag/AgCl, respectively, were identified to correspond to the processes of oxidation (Fe^2+^ → Fe^3+^) and reduction (Fe^3+^ → Fe^2+^). The suggestion is that the capacitance of MnFCN electrodes is a result of the presence of both Mn and Fe ions. The GCD curve’s configuration confirms the course of the redox reactions on the surfaces of the fabricated electrodes. According to the results obtained through the GCD analysis, the specific capacitance was determined to be 327.5 F/g at 1 A/g (Figure 4e). 

Electrochemical measurements of MnFCN/NF as a cathode for a hybrid zinc-ion supercapacitor were obtained using a 1 M ZnSO_4_ solution as an electrolyte. In Figure 4c, the CVA curves show two pairs of peaks in the range from 0.0 V to 1.2 V vs. Ag/AgCl. The CVA curves exhibited negligible deformation, even when examined at multiple scan rates, which suggests that MnFCN/NF displayed exceptional reaction kinetics when undergoing rapid charging and discharging. Figure 4f shows the GCD curves of a MnFCN/NF hybrid zinc-ion supercapacitor at different currents. The analysis of the discharge curves demonstrates that at a current density of 0.1 A/g, the specific capacity values amounted to 230 F/g. 

**Figure 5:**
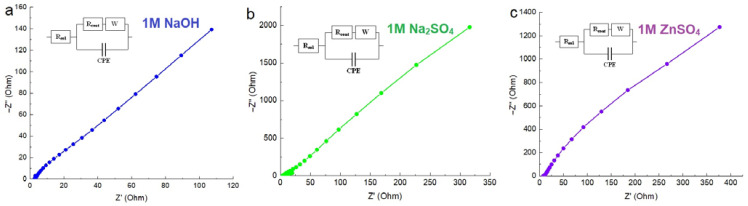
EIS spectra of MnFCN/NF in (**a**) 1M NaOH, (**b**) 1M Na_2_SO_4_, and (**c**) 1M ZnSO_4_ electrolytes.

Electrochemical impedance spectroscopy (EIS) was employed for the purpose of investigating the charge transfer behavior of the MnFCN electrode material. Figure 5 shows the EIS spectra of the MnFCN/NF electrode in 1 M NaOH (a), 1 M Na_2_SO_4_ (b), and 1 M ZnSO_4_ (c) aqueous solutions. The findings indicate that the decreased semicircle size, reduced intercept, and greater vertical line towards the low-frequency side of the samples imply a more prominent mass transfer limit and diminished interfacial charge resistance. These observations suggest that the sample exhibited enhanced conductivity and mass transport characteristics in all electrolyte types. 

A comparison of the specific capacities of electrodes based on PBAs in different electrolytes synthesized using different methods is shown in Table 1. The results obtained in this study demonstrate the proximity and, in certain instances, superiority of the presented values in comparison to the characteristics of samples produced through alternative synthetic methods. The superior performance exhibited by the MnFCN cathode may be attributed to its layered structure, which greatly facilitated the adsorption/desorption and diffusion of electrolyte ions when subjected to rapid charging–discharging cycles. Furthermore, the agglomerates with spherical morphologies, which were acquired through the incorporation of two-dimensional nanosheets, offered a greater number of active sites for the purpose of electrochemical reactions. Consequently, this enhanced the specific capacitance value. 

## 4. Conclusions

The present investigation represents a novel achievement, as it reports the successful synthesis of a Mn_3_[Fe(CN)_6_]_2_·nH_2_O thin film composed of a set of two-dimensional nanosheets utilizing the SILD technique. A notable improvement in the specific capacitance was observed, reaching 1032 F/g when operating at 1 A/g with an aqueous alkaline 1M NaOH electrolyte. Moreover, the discharge capacitance values of 327.5 F/g at 1 A/g and 230 F/g at 0.1 A/g were obtained in 1M Na_2_SO_4_ and 1M ZnSO_4_ aqueous solutions, respectively. These results show that Mn_3_[Fe(CN)_6_]_2_·nH_2_O is a prospective candidate for practical applications as a supercapacitor in both alkaline and neutral media and, in particular, as a cathode material for a hybrid Zn-ion supercapacitor.

## Figures and Tables

**Table 1:** Comparative table of electrochemical characteristics of electrodes based on PBAs.

Electrode Material	Electrolyte	Current Density (A/g)	Specific Capacitance (F/g)	Refs.
MnHCF/GO	1.0 M Na_2_SO_4_	0.3	279.3	[25]
PB/rGO films	1.0 M Na_2_SO_4_	0.3	286.0	[26]
CoHCF	0.5 M Na_2_SO_4_	1.0	284.0	[27]
CoHCF	0.5 M Na_2_SO_4_	1.0	250.0	[28]
ZnCo-PBA@α-Co(OH)_2_	1.0 M KOH	1.0	423.9	[29]
CoS_2_-derived Co-Co PBA	2.0 M KOH	1.0	936.0	[30]
K_2.25_Ni_0.55_Co_0.37_Fe(CN)_6_/CNTs	4.0 M KCl	0.2	600.0	[31]

## Data Availability

Not applicable.
